# Mississippi Valley Dental Association

**Published:** 1874-06

**Authors:** 


### DISCUSSIONS OF THE THIRTEITH ANNUAL MEETING OF THE MISSISSIPPI VALLEY DENTAL SOCIETY IN JOINT SESSION WITH THE MISSOURI DENTAL SOCIETY, HELD IN ST. LOUIS, MARCH, 1874.

### 1ST DAY.

Dr. W. H. Eames called the meeting to order at 11 o'clock.

The exercises were opened with prayer, by the Rev. Dr. Coan.

Dr. Taft, of Cincinnati, not being present; it was moved by Dr. Leslie, and seconded by Dr. Goodrich, that the address of welcome and the response thereto be deferred until the afternoon session. Put to the vote and carried. The Chairman then declared the meeting open for the discussion of the first subject on the programme, "The treatment of deciduous teeth." No person rising to a discussion of the subject, it was passed; and the second subject, "Is gold the only material to be used in the conservative treatment of the teeth," announced.

Dr. Morgan: I presume the meeting, generally, is open for discussion. Let us have the experience of the profession, and their views upon the first subject. A paper is not absolutely necessary.

I have very little to say upon this subject, although, I regard it as one of very great practical importance. A very large number of our patients come into our hands when but little advanced in years, and the question arises to every practitioner, What shall I do with these deciduous teeth? As a general rule I think they should be treated upon the same general principles which govern the practice of the profession in other branches. I have but little to add in the way of special treatment; still there are some important facts connected with the treatment of deciduous teeth. In the first place, the dentine of these teeth seems less sensitive than that of the permanent set, and it is less liable to be acted upon by corrosive agents. Filing may be used more frequently than in the case of the adult teeth. As a general rule, when the pulps of the deciduous teeth are devitalized, they should be removed from the fear of abscess or inflammation extending to the germ of the permanent teeth. Of course, there are exceptions in all cases. A great number of permanent teeth are injured by permitting devitalized teeth to remain in the mouth causing abscess or inflammation extending to the crown of the permanent tooth, and doing injury to the enamel at that time which is never repaired. I am familiar with many cases of defective permanent teeth where the defective formation of the enamel can be traced to no other cause. I have watched some cases and removed deciduous teeth when they have caused inflammation in the maxillary. I have traced to this cause more than one case of exfoliation of the alveolar processes, where the partially formed crown of the permanent tooth has been brought away with the temporary tooth. The decay of the temporary teeth should be stopped as we stop that of the teeth of the adult subject by removing the decay and filling with some substance that will preserve it until nature casts it off. He did not consider that after a tooth was devitalized any great amount of absorption of its material took place.

I would like to hear from Iowa. I think I have heard some Iowa man say, that the dentist who removed deciduous teeth should be indicted for malpractice.

Dr. Kulp: I represent Iowa to some extent. If the gentleman refers to anything emanating from me, I don't think I am the man. I don't think I ever made such a remark as that, still I should be willing to take issue with some points in his position. I consider some things that he has advanced as erroneous. I do not consider the removal of the temporary teeth good practice. My practice has always been to save children's teeth as far as possible, for various reasons. Now, while inflammation from the deciduous teeth may possibly injure the permanent teeth, I have never in my life been able to discover and can not now see why local inflammation should injure the permanent teeth, while the tooth is enveloped in a bony socket. Here is another point. It is a very important period in the child's life, that of shedding the teeth. The child needs all its power of mastication to prepare food for the stomach. It is also the time when the child learns to talk, and I think it is very important that the deciduous teeth should be preserved for the purposes named. I think the subserving of that purpose counter-balances any injury to the permanent teeth. I have just been to a case I saw yesterday, a little boy about four years old with his central incisors decayed, the nerves dead and his face terrible swollen. He was sent to me by a physician with peremptory orders to extract these teeth. I thought that really it would not be right to do so, and treated them as I would permanent teeth. I think it is very important to keep these for, perhaps, a year or a year and a half longer as the child is begining to talk. When the permanent teeth advance I shall remove the deciduous teeth, but until that time comes I shall certainly leave them. "Doctor Kulp gave another case of a little girl of his, whose teeth at the age of five commenced to decay very rapidly, he devitalized the nerves and filled the teeth and thought from his observation in this case that absorption of the root of a devitalized tooth did take place. He would certainly consider the man who pulled deciduous teeth promiscuously at any time of life indictable, he should be scolded any way and possibly taken up for malpractice.

Dr. Forbes inquired how the extraction of decayed deciduous teeth would injure the permanent set.

Dr. Kulp replied, it might change the position of the permanent teeth. If a child came with ulcerated teeth he would extract them, because of irritation; but if the crown was there, so that it could be made useful, he would try and save the tooth.

Dr. Morgan: "I said there was danger that an abscess formed upon a deciduous tooth might injure the coming tooth. I did not state that it would injure the perfectly formed enamel. The injury is done at the time the lime salts are being deposited. Dr. Kulp has spoken of a case where he had restored such teeth to perfect health.

I hold there never was a case yet where there had been abscess that perfect healthiness of the parts had been restored. There is always more or less diseased action around the root upon which the abscess is formed. The tooth has been devitalized, hence neither it nor the periosteum can be restored to perfect health. I admit the absorption to a very small extent, but it has not removed the roots of the teeth in any case I have observed.

Dr. Spaulding, being called for, remarked, that there were several physiological points involved in the question before them. He would like to reply to the question just asked, as to whether it was good practice to fill the roots of deciduous teeth, most decidedly yes, if twenty years' experience in filling the roots of such teeth with almost universal success was any criterion from which a judgment could be formed. He filled them with gold, and considered it of the utmost importance to retain the deciduous teeth as long as they could be retained in a state of tolerable health. The doctor then spoke of the connection between exanthematous diseases and defective teeth. All skin diseases occuring during the formation of the enamel of the teeth were marked more or less with imperfect condition of this tissue. He considered the absorption of the material of the temporary teeth and the formation of the enamel of the permanent teeth as two distinct processes.

Dr. Kulp inquired, may not the cause that originates these eruptive diseases be the cause of the imperfect formation of enamel?

Dr. Spaulding: I think that would only account for the general character of the enamel, not for the condition of it in special localities.

Dr. Morgan thought the cause was not always constitutional.

Dr. Hunter raised the question whether the bone material proper of the roots of the deciduous teeth went to help in the formation of the roots or any portion of the permanent teeth. If so, it would be positively wrong to extract the deciduous teeth before there was a positive necessity for it.

Dr. Morgan had no theory upon that subject, but was of the opinion that nature never appropriated the same material more than once.

Dr. Dean, being called for, thought they were not discussing the right subject, the subject before the association was the treatment and care of the deciduous teeth. He knew nothing new to add to this subject.

Dr. Cushing was glad the discussion had called out some of the points given, especially the remarks of Dr. Spaulding. From his observations in a large number of cases he was inclined to think Dr. Morgan attributed more injury to the inflammation of the deciduous teeth than was chargeable to it. Erom his recollection of the period at which the enamel of the second set of teeth was formed, he considered that in the majority of cases in which such inflammation could ensue the enamel would be pretty completely formed.

Dr. Rivers wanted to know if the case of defective or imperfect enamel met with in the molar teeth and very frequently in the wisdom teeth, which were not erupted till the age of twenty-five, were attributable to the same cause.

Dr. Morgan said they were not.

Dr. Black wished to express the idea that the blocking up of the dental tubuli with sulphuret, which would occur when the roots were left open, would interfere with absorption, and probably induce disease, which might be prevented by filling the roots.

Dr. Rehwinkle, remarked, that if there was any case where the operation of filling the roots was admissible it certainly was in the case of deciduous teeth. It would prevent the formation of abscesses after the operation, but not entirely so; where the teeth were devitalized a double duty was thrown on the periosteum, and which was liable to lead to disease, if exposed to the contagion of diseased matter. He believed in filling with cotton saturated with creosote or carbolic acid. Very few of them were anxious to fill children's teeth with gold. They might do it occasionally, but experience proved there was neither money nor pleasure in the task.

Dr. Keely replied, that they were limited with regard to the saving of children's teeth, owing to the impracticability of the task in some instances. It was dfficult to control them unless you had a good supply of magnetism. He considered himself possessed of as much patience as any man since the days of Job, and he did not always fill children's teeth with gold. He had used gold, tinfoil, oxychloride of zinc and amalgam. He was almost afraid to say "amalgam." The speaker then gave a case of his daughter, nine years old, whose temporary molars had decayed at the age of two and a half years. The superior one he filled with gold, one of the inferior ones with tinfoil, the other with amalgam. She had one of those teeth yet.

Dr. Wells then gave the particulars of the case of a little girl two years old, whose teeth were falling out unaccountably fast, and asked for an explanation of the same.

The meeting was then adjourned to meet at 2 o'clock.

AFTERNOON SESSION.

After the opening exercises, the discussions on the treatment of deciduous teeth were resumed.

Dr. Taft: I don't know that I ought to say anything upon this subject not having heard all the discussion. But from what I heard of the morning's discussion, it seems to me that those who engaged in it confine themselves too much to points of minor importance, I think the subject would have been better denominated, "The management of deciduous teeth." The discussions upon the subject thus far seem to refer to the teeth, only after they have become decayed or diseased, but my understanding of the subject is, that we are dealing with the teeth during their health as well as after their decay and disease, and I consider that to understand what may be done to preserve those organs in a healthy condition is of more importance than to know what to do after they have become diseased, I think we are disposed to limit ourselves too much to the treatment of disease and ignore hygiene. The question reaches far back, if we should follow it up, but it should be considered here, at least, so far back as the growth and development of the temporary teeth, and the question to be answered seems to me to be. "How can we secure for a child a good set of temporary teeth? To do this we must use an enlightened judgment and knowledge, and here, as in many other things some of us possess more knowledge than we use. If we gave mothers hygienic rules and directions for the rearing of children and taking care of the temporary teeth, much would be accomplished and a great deal of dental disease prevented, and especially by careful attention given to those teeth during their period of eruption. I believe the temporary teeth can be preserved in all ordinary cases, (unless some unfavorable systemic conditions exist or occur) until the time of their throwing off by nature. I have had children brought to me with their teeth covered with deposits of a vitiated character, and yet their mothers did not know but what the teeth were in a perfect condition.

There are cases of this kind continually coming before us. There are of course some exceptions, but it ought to be thoroughly installed into the minds of all mothers and fathers too, that it is just as reprehensible to permit their children to have dirty teeth, as to be unclean in any other respect.

As to filling the roots of the temporary teeth, I do not believe it to be good practice; I have not resorted to it for years and do not think I shall ever do so. In those that are devitalized the pulp should be removed and all the debris excluded as far as possible, every acrid accumulation should be neutralized and nothing left to act deleteriously upon the living tooth beneath. He regards the use of carbolic acid as improper and recommended that instead of filling with gold or any metallic substance, the cavity be simply kept closed, so as to prevent the lodgment and intrusion of foreign substances. In regard to the absorption of the roots of the temporary teeth, there is no pressure excited by the tooth in process of formation and eruption, below or above: and it is my opinion that the material absorbed is not re-appropriated, but is effete matter and is thrown out of the system.

Dr. Crouse remarked upon the want of unanimity among the speakers and expressed himself as unable to see what injury to the permanent teeth was done by the filling of the deciduous ones. He thought a little common sense would greatly aid in the discussion on hand.

Dr. Dean quoted Dr. Lionel Beale to show that organic material could be re-appropriated in the case of the muscles and in the formation of bone. In the latter instance, material was taken from one part of a bone and deposited in another. He thought there was certainly sufficient analogical evidence to support the opinion that a similar process could take place with regard to the teeth.

Dr. Rehwinkle: On motion of Dr. Rehwinkle, discussion subject was closed.

SECOND SUBJECT.

The next subject: "Is gold the only material to be used in the conservative treatment of the teeth?" was announced, but no one seemed willing to commence the discussion.

Dr. Taft said the quickest way of disposing of the subject was to simply answer "No."

THIRD SUBJECT.

Dr. H. A. Smith read a paper on the next subject, "Will legislative enactments prevent those who lack the necessary qualifications from entering the dental profession?" He discussed the subject philosophically, and argued in favor of a final examination of all dentists by a Board of Examiners before they are permitted to practice. A law similar to the one in Ohio was what was needed.

Dr. Crouse considered legislation as one means by which the end aimed at could be accomplished. In Illinois they had a bill before the legislature which they hoped would pass. It did not interfere with any man practicing in that state at the present time, but after the passage of the bill, no dentist could come in from another state without standing an examination or producing a diploma from some respectable dental college, and no young man in the state could enter the profession without becoming a graduate of a dental college. Dr. Crouse animadverted very severely on the practice of some dentists in taking ignorant boys as studants into their office, all such boys learned, was to do the dirty rubber work and then they were turned loose upon the community, and it was something to be wondered at, if even after eight or ten years of slaughtering their patients' teeth they became decent dentists. They had framed and adopted a by-law not to allow any member to take a student for less than three years, and not then without a certain degree of assurance that he would become a graduate before he entered into practice, and if they could enforce that law they would work some good.

Dr. F. H. Rehwinkle believed that legislative enactments would prevent many incompetent persons from entering the profession. He spoke of the custom in the old country, where boys were indentured to professions and made to serve a certain number of years before receiving a diploma or a certificate, and illustrated his remarks by a description of the different grades of the medical profession in Germany, where each was limited to a certain class of work according to his degree or qualification.

Dr. H. A. Smith said the cry of special legislation would be raised when any law of the kind was enacted. He believed it would be hard to prosecute under the law, for the reason that a man, no matter how poor a dentist, will have friends whom he can get on a jury, and who will invariably acquit him.

He did not agree with Dr. Crouse, in requiring a man to be a college graduate before he could practice, but believed many self taught men would pass the examination better than those who were college trained.

Dr. Keeley said that he had heard it said concerning some applicant for certificate from the board of examiners in Ohio, that many of those who had failed once had gone home, passed six months or a year in study and again presented themselves and failed, who had gallantly come up for the third time and passed. Speaking of the bill as passed in Illinois, he believed it was so drawn up that its provisions could be enforced, and he was glad the Illinois dentists had not asked for too much, and considered that a law which produced the results he had mentioned was doing much good.

Dr. C. S. Smith, of Springfield, Illinois, said in his State they had been trying for the last four years to get the legislature to pass such a law, and had about succeeded. The cry that the law would create a monopoly on the part of the big dentists was raised, and it was hard to get the members to see that the protection of the people was what they aimed at.

Dr. Taft thought much good had been done in Ohio by the law. Dentists there did not take boys into their offices to learn the rudiments, and then send them forth to business on their own account. It was known, however, that several who went before the board of examiners and could not pass an examination, had gone out of the State and set up elsewhere. He didn't know where they went to. There might be some of them here in St. Louis, and he was certain a few had gone to Kansas and other States.

Dr. Bancroft, of the province of Ontario, Canada, asked permission to say a few words, which was granted. He said they had a law in Canada which proved efficient. Dentists were divided into three classes. Those who studied five ye..rs were entitled to a certificate without an examination; those who studied a portion of five years could reeeive a certificate on examination, if competent; the last class embraced young men desirous of entering the profession—these could not enter an office without matriculating. Seven examiners of ma triculation were appointed. In the province not one is practicing who has not a certificate.

In reply to a question, the doctor stated that any person from the United States or from any foreign country must first appear before the board of examiners and receive a certificate, before he can engage in the duties of the profession.

Dr. Richardson spoke of the efforts that had been made to pass a Dental Bill in Indiana, and humorously remarked that he believed the operation of the law in Ohio had driven all the incompetent dentists out of that State into Indiana, for they were quite overrun with them.

The Chairman then stated, the Executive Committee had reported the order of business for the next morning to be "clinics," at College Infirmary, No. 904 Chestnut street, Drs. Morgan, Crouse, Griswold and Taft to conduct the exercises. The same to be followed by a joint session in the afternoon.

Adjourned to 7:30 P. M.

NIGHT SESSION.

The fourth order of business:

"Hasty operations, or trotting them through," was passed over, it being understood that Dr. Keeley had a paper to read on that subject, and that gentleman not being present.

"What are the best materials for filling the roots of teeth?" was then taken up.

Dr. Dean called for Dr. Morrison's views.

Dr. Morrison: I have nothing further to add to what I have already submitted on this subject.

The Chairman: As the Doctor has made his remarks through the medium of the Journal I think some one should sustain or oppose him.

Dr. Dean: I have not had time to read that article fully, I believe it shows that in his opinion gold is the best material for filling the root canals of teeth; in some special cases, however, where the time involved in the operation would be too great, and the operation would be too expensive, perhaps something else would be a good substitute, whereby the operation would be rendered less tedious and, perhaps, quite as good for the root canal in the front teeth.

In the upper incisors, canines and biscupids, I would prefer gold, the heavier numbers of gold, say No. 20. wound upon a sm oth straight plugger; this does not increase the size of the plugger but carries the foil as it were upon the broach just where you want it. Upon the end of this little instrument the foil can be carried in and then withdrawn and another portion carried in until the cavity is thoroughly filled; in that way you can introduce the gold into these root canals more rapidly than in the larger cavities and with greater certainty. I believe some gentlemen have been using lately, spice wood saturated with carbolic acid or creosote. One of the most prominent dentists in Philadelphia wrote to me that he filled most of his root cavities with spice wood saturated with creosote. I can see no objection to this mode of filling the more tortuous canals; the greatest objection is, it might be passed through the canal and become an irritant, but I don't see why this can not be prevented by measurement, I certainly should not fill any of the front teeth with cotton saturated with creasote, because there you can introduce gold perfectly, and these teeth are important teeth to save; as to os-artificials I presume they are all good at times and in their right place.

Dr. Morrison wished to make an explanation concerning his article in the Journal. "The method spoken of by myself in that article is to fill these roots with pure gold wire, to have different sizes of wire graded according to the canal you wish to fill, file the wire until it fits the canal, you thus have the privilege of trying it on the root; if it goes too far through the foramen you can take it out and try a larger size; the objection to foil roll is, you may roll it one thickness too much or too small. When your wire just fits the canal you cut a niche in it where you wish to break it off and then tap it well in, one little rotation of the instrument leaves it there for ever.

Dr. Cushing endorsed what Dr. Dean said with regard to the use of gold in canals which were easy of access, but was inclined to think some other material might be used where it could be used easily. He had seen dissolved gutta-percha recommended by some for the filling of small tortuous canals which could not be thoroughly filled with gold. He had used that method to some extent himself and found it worked successfully. He would prefer dissolved gutta-percha to oxicloride of zinc, because it could be more easily introduced into the canals. He had no objection to urge against cotton or silk saturated with creosote.

Dr. McCord: Some three or four years ago some gentleman, I think it was Dr. Knapp, left with me some preparations of gutta-percha manufactured by S. S. White, intended to be used for filling teeth temporarily, I made several attempts but could never do anything with it. Afterwards I had occasion to use it again and after a few attempts I succeeded, since which time I don't want anything better. After preparing the root, I cut off the stopping with the wedge cutters in little pieces the length required, in a conical shape hardly a quarter of an inch long, I have several of these pieces, and after drying out the cavity I wipe it out with creosote, then warm one of these little cones in the flame of the lamp and lay it in the cavity with a gold instrument, it won't go through and being warm will take the form of the canal, then put in another piece till the cavity is filled; it is the most expeditious method; I would not use wood, you can put this preparation in any kind of a crooked canal and push it and make it fill the cavity perfectly. Another substance is a varnish made of resin dissolved in alcohol; this is used with cotton, the cotton being soaked in it, and shortly after introduction it becomes quite hard while it is indestructable.

Dr. Rivers thought they were leaving out of the question the condition at the time of filling, and also that they had overlooked the short period of time which had elapsed since making some of these fillings.

Time only would reveal whether the operations were successful or not. The filling that would answer for one tooth might be the very worst possible for another. The doctor reverted to the various substances used and to the various modes of filling and the different periods of time elapsing between the preparation of the cavity and remarked that great success was claimed for every case. They all knew that if they had a tooth under treatment in which the pulp was so badly exposed that it must be devitalized, it would not do to attempt to remove that pulp immediately; if the attempt was made they would only succeed in bringing it away in fragments. He would simply state from his own observation that with teeth in this condition it didn't much matter whether they were filled with gold, gutta-percha, orange-wood, soaked in creosote or collodion. He thought under such circumstances the cavity would be best filled with oxichloride of zinc. It had always been his custom where the pulp of a tooth was devitalized to wait a considerable length of time according to the teeth before completing the filling, this would allow sufficient decomposition of the pulp to take place, then they could take the nerve broach, carefully entangle it in the pulp, and, in the majority of cases, the pulp can be brought away entire in the incisor teeth; where access to the root canal is direct he would get at the pulp by reaming out the cavity and under favorable circumstances, he said, to filling the teeth at once; if that could not be done he would introduce cotton soaked in carbolic acid, and allow it to remain over, say twenty-four to thirty-six hours, and closing up the cavity with a different material; when that cotton was removed they would be surprised what it would bring away when the cavity was perfectly clean. He did not care how they filled that tooth, he thought that gold was the best for the front teeth; it was so genteel and nice; he usually used gold in the form of wire or rolled cylinders, but the shape in which the gold was used was of very little consequence; in his opinion the preparation of the cavity was the important item upon which success depended.

